# Inhibition of Glutamate Dehydrogenase as a Potential Strategy to Modulate Intrahepatic Cholangiocarcinoma Cell Metabolism

**DOI:** 10.3390/biom16030449

**Published:** 2026-03-17

**Authors:** Anna Santarsiero, Ilaria Pappalardo, Alessandro Santarsiere, Ernesto Santoro, Marisabel Mecca, Antonio Evidente, Pierluigi Reveglia, Lucia Lecce, Federica De Carlo, Carlo Calabrese, Vittoria Infantino, Stefano Superchi, Simona Todisco

**Affiliations:** 1 Department of Health Sciences, University of Basilicata, 85100 Potenza, Italy; anna.santarsiero@unibas.it (A.S.); vittoria.infantino@unibas.it (V.I.); 2 Department of Basic and Applied Sciences, University of Basilicata, 85100 Potenza, Italy; ilaria.pappalardo8@gmail.com (I.P.); alessandro.santarsiere@unibas.it (A.S.); ernesto.santoro@unibas.it (E.S.); 3 Laboratory of Preclinical and Translational Research, Centro di Riferimento Oncologico della Basilicata (IRCCS-CROB), 85028 Rionero in Vulture, Italy; marisabel.mecca@crob.it (M.M.); federica.decarlo@crob.it (F.D.C.); carlo.calabrese@crob.it (C.C.); 4 Institute of Biomolecular Chemistry, National Research Council (CNR), 80078 Pozzuoli, Italy; antonio.evidente@unina.it; 5 Department of Clinical and Experimental Medicine, University of Foggia, 71122 Foggia, Italy; pierluigi.reveglia@unifg.it (P.R.); lucia.lecce@unifg.it (L.L.)

**Keywords:** glutamate dehydrogenase, punicalagin, ellagic acid, intrahepatic cholangiocarcinoma, pomegranate waste extract, NF-κB

## Abstract

Cholangiocarcinoma (CCA) is a rare malignancy of the biliary tree with increasing global incidence and mortality and limited therapeutic options. Intrahepatic cholangiocarcinoma (iCCA) metabolism exhibits enhanced glycolysis, oxidative phosphorylation, and glutamine utilization. In this study, we investigated the therapeutic potential of targeting glutaminolysis in iCCA, identifying glutamate dehydrogenase (GDH)—which converts glutamate to α-ketoglutarate—as a key metabolic hub. We evaluated the effects of pomegranate waste extract (PWE), a by-product of industrial pomegranate juice production, on cell viability, proliferation, migration, ATP production, and extracellular acidification in CCLP1 cells, an established iCCA model. Our results are consistent with an altered cellular energy metabolism. We further assessed GDH enzymatic activity, expression, and transcriptional regulation in the presence or absence of PWE and its major components, punicalagin and ellagic acid. GDH expression was downregulated by PWE in a dose-dependent manner through inhibition of NF-κB signaling, revealing a new mechanistic link between NF-κB and GDH. In addition, GDH enzymatic activity was dose-dependently inhibited by PWE, as well as punicalagin and ellagic acid. Notably, punicalagin was identified as a novel competitive inhibitor of GDH. Overall, these findings provide the first evidence that modulation of glutaminolysis through GDH targeting impairs iCCA cell growth and metabolism, supporting GDH as a promising metabolic target. This study highlights pomegranate-derived compounds as potential leads for the development of adjunctive or preventive strategies in intrahepatic cholangiocarcinoma.

## 1. Introduction

Cholangiocarcinoma (CCA) is a rare cancer that originates from the biliary tree. CCA is classified into intrahepatic (iCCA), perihilar (pCCA), and distal (dCCA); the latter two are collectively referred to as extrahepatic cholangiocarcinoma (eCCA). The incidence and mortality are increasing globally, with 10% of iCCA cases, about 50% of pCCA cases, and 30–40% of dCCA cases [1]. In the early stages, CCA is asymptomatic; therefore, when it is revealed, the diagnosis is unfavorable with a poor prognosis and a 5-year survival rate of around 7–20% [2]. Surgical resection is the primary treatment for CCA, typically followed by adjuvant therapy. When CCA is unresectable, only palliative treatment options are available, including cisplatin/gemcitabine-based chemotherapy combined with programmed death-1 (PD-1)/programmed death-ligand 1 (PD-L1) inhibitors [3]. However, response rates to these treatments are highly variable, and overall survival remains poor [4].

Recently, new therapies have been approved for patients with specific molecular alterations who experience disease progression after first-line treatments. Notably, mutations of the metabolic enzyme isocitrate dehydrogenase (IDH), which catalyzes the conversion of isocitrate into α-ketoglutarate (α-KG) [5], have been found in 10–20% of iCCA cases and about 1% of eCCA cases. Treatment with Ivosidenib, a specific reversible inhibitor of mutant IDH1, reverses tumor cell behavior, making this inhibitor a therapeutic option for advanced CCA with IDH1 mutation [6]. This is supported by the results of the ClarIDHy phase III clinical trial (NCT02989857), which demonstrated significantly improved progression-free survival and a favorable safety profile in patients with IDH1-mutated CCA.

The metabolism of CCA is characterized by a high glycolytic flux, with many glycolytic enzymes upregulated, such as hexokinase II [7] pyruvate kinase M2 (PKM2) [8], and lactate dehydrogenase A (LDH-A) [9]. CCA cells are also characterized by a high OXPHOS, which addresses the metabolism toward mitochondrial respiration [10]. Interestingly, the inhibition of the respiratory chain with both phenformin and metformin results in an efficient reduction of cancer cell proliferation [11,12]. The pentose phosphate pathway (PPP) was also found to be activated in CCA cells to supply nucleotides and NADPH for antioxidant defenses [13]. Notably, it was demonstrated that the transcriptional factor Nuclear factor Erythroid 2-related factor 2 (NRF2), which regulates the expression of many enzymes of PPP, is activated in CCA cells, leading to a decrease in reactive oxygen species (ROS) and oxidative stress in cancer cells [14].

Furthermore, CCA cancer cells exhibit a dependency on L-glutamine as a nutrient source. L-glutamine participates in different cellular processes, including nucleotide biosynthesis, glutathione production, and epigenetic modifications, and acts as a donor of the carbon skeleton [15]. In eCCA cells, L-glutamine deprivation reduces chemoresistance [16], while in iCCA cells, simultaneous inhibition of L-glutamine and glucose uptake overcomes cisplatin resistance [17]. In CCA cells, L-type amino acid transporter 1 (LAT1) downregulation or inhibition decreases proliferation and migration of CCA cells, opening up a potential therapeutic strategy [18,19,20]. The involvement of the glutaminolysis pathway is highlighted by the overexpression of mitochondrial glutaminase (GLS1)—which converts L-glutamine into L-glutamate and ammonia—in iCCA cells [21] and of glutamate dehydrogenase (GDH)—which converts glutamate to α-ketoglutarate (α-KG)—in eCCA cells [22]. Inhibition of GLS1 or silencing/inhibition of GDH has been proposed as a promising strategy to enhance cancer therapy [22,23,24].

Two isoforms of GDH exist in humans: hGDH1 and hGDH2 encoded by the *GLUD1* and *GLUD2* genes, respectively, which show high similarity in amino acid sequence (97%) but differ in tissue expression. In fact, hGDH1 is particularly expressed in the liver, whereas hGDH2 is expressed in neural and testicular tissues but not in the liver [25]. Considering that several plant-derived compounds have shown an .inhibitory effect on GDH [23,26], the present study investigated pomegranate-derived compounds as novel GDH inhibitors.

*Punica granatum* L. (Punicaceae), commonly known as pomegranate, is rich in phytochemical compounds, such as punicalagin, gallic acid, and ellagic acid. Extracts from different parts of the pomegranate fruit exhibit several biological effects, including antimicrobial, antioxidant, anti-inflammatory, and anticancer activities [27,28]. In breast cancer models, pomegranate-derived compounds have been shown to have antioxidant and pro-apoptotic properties [29], modulate gene expression [30], and inhibit angiogenesis, proliferation, and growth while increasing apoptosis. Interestingly, the valorization of pomegranate waste has enabled the extraction of these valuable bioactive compounds, which also exhibited cytotoxic and genotoxic effects on liver cancer cells [31,32].

In this context, we investigated the biological effects of pomegranate waste extract (PWE) on the metabolism of CCLP1 cells, a model of iCCA. The PWE was obtained by ethanolic extraction of exhausted peel, a by-product of industrial pomegranate juice production, selected both for its high polyphenolic content and to promote the valorization of agricultural waste. Initially, we assessed the impact of PWE on cell proliferation, migration, and metabolic profile. We then examined its effects on the expression and activity of hGDH1, a target not previously explored in iCCA. Following the identification of GDH inhibition, we investigated the most abundant components of PWE—punicalagin and ellagic acid—to identify the compounds responsible for this effect. Overall, our findings highlight GDH as a promising metabolic target in iCCA and identify novel GDH inhibitors.

## 2. Materials and Methods

### 2.1. Plant Material and Extraction

The ‘Wonderful’ variety of pomegranate (*P. granatum* L.) used for extraction was an industrial pressing residue provided by Mercorella agricultural company, located in Scanzano Jonico, Basilicata, Italy. For the extraction process, 500 mg of the exhausted pomegranate peel was mixed with ethanol at a solid-to-liquid (S/L) ratio of 1:2 (*w*/*w*) and stirred at 70 °C for 130 min. Following extraction, the sample was centrifuged at 10,000 rpm for 20 min at room temperature. The resulting ethanol extract was filtered through a syringe septum (pore size 0.45 μm) and concentrated under reduced pressure. The reddish solid residue obtained was designated as PWE (Pomegranate Waste Extract) and will be referred to as such hereafter. PWE was subjected to LC-MS/MS analysis of phenolic acids and flavonoids. Subsequently, PWE was dissolved in distilled water and diluted again in water to achieve the desired final concentrations. Concentrations are reported as μg/mL or ng/mL depending on the experimental range for cell-based experiments and GDH activity experiments.

### 2.2. Chemicals and Standards for LC-MS/MS Analysis

Reagents used for the analysis, including LC-MS grade methanol (≥99.9%, LiChrosolv^®^), formic acid (98–100%, EMPROVE^®^ ESSENTIAL), quinaldic acid, and punicalagin, were purchased from Sigma-Aldrich (Darmstadt, Germany). The Phenolic Acids and Alcohols Standard Mixture—V2 and the Flavonoids Standard Mixture—V2 were obtained from the MetaSci library (https://www.metasci.ca/ (accessed on 24 February 2025)). These standard mixes were used for peak identification, method development, and calibration curve construction.

### 2.3. LC-MS/MS Analysis of Phenolic Compounds

The LC-MS/MS system consisted of a UHPLC (Nexera Series LC-40, Shimadzu, Kyoto, Japan) coupled to a triple quadrupole/linear ion trap tandem mass spectrometer (QTRAP 4500, AB Sciex, Framingham, MA, USA) equipped with a Turbo V ion source. Instrument control, data acquisition, and processing were performed using the associated Analyst 1.6 and Multiquant 3.0 software. The LC separation was carried out on a Gemini C6-Phenyl 110Å column (50 × 2.0 mm, particle size 5 µm) from Phenomenex (Torrance, CA, USA). Elution was performed at a flow rate of 700 µL/min with water containing 0.1% (*v*/*v*) formic acid as eluent A and methanol containing 0.1% (*v*/*v*) formic acid as eluent B. Two slightly different gradient chromatographic methods were developed for phenolic acids and flavonoids. For phenolic acids after a column equilibration time of 3 min: 0–5 min, from 2 to 30% of phase B; 5–7 min, 30–100% of phase B; 7–9 min hold the phase B concentration; 9–10 min back to initial condition 2% of phase B. For flavonoids, after a column equilibration time of 2 min: 0–5 min, from 2 to 30% of phase B; 5–7 min, 30–100% of phase B; 7–9 min hold the phase B concentration; 9–12 min back to initial condition 2% of phase B. The column oven was set to 40 °C, and 2 mL of the samples were injected. Q1 resolution was adjusted to 0.7 ± 0.1 amu fwhm for MRM, referred to as the unit resolution. Q3 was also set to the unit resolution in MRM mode. Two scheduled MRM (sMRM) methods were developed, one for phenolic acids and punicalagin containing 44 metabolites, and one for flavonoids containing 38 metabolites, for 82 polyphenols. Examples of standard chromatograms are reported in Supplementary Figures S1 and S2. MS analysis was carried out in negative and positive ionization mode using an ion spray voltage of ±4500 V. The nebulizer and the curtain gas flows were set at 35 psi using nitrogen. The Turbo V ion source was operated at 350 °C for phenolic acids and 450 °C for flavonoids. The Ion Source Gas 1 and 2 were both set at 40 psi. Suitable MRM transitions were selected for the targeted metabolites and Quinaldic acid, which were used as the internal standard (IS). The compound-dependent parameters for metabolites and IS were optimized using the manual optimization protocol in tuning mode. The Q1 mass, the Q3 transition, the best parameters, and the retention time are reported in Supplementary Table S1. Quinaldic acid was spiked to each sample solution at a final 0.5 µg/mL concentration. The Quinaldic Acid Area values were utilized to monitor the quality and robustness of the analysis.

### 2.4. LC-MS/MS Methods Validation

LC-MS/MS method validation was conducted by analyzing calibration curves, limits of detection (LOD), and limits of quantification (LOQ). LOD and LOQ were calculated as the amount of analyte that gives an S/N ratio of 3 for LOD and 10 for LOQ. Independent calibration curves were constructed for both phenolic acids and flavonoids using the serial dilution method. The calibration curves were generated by plotting the metabolite area of the selected sMRM transition against the concentration of the metabolite solution. The linearity range of each metabolite was assessed using eight calibration points, and each point was analyzed in triplicate. The linearity range, curve equations, LOD, and LOQ are summarized in Supplementary Table S2.

### 2.5. Cell Cultures

Established human CCA intrahepatic (CCLP1) and extrahepatic (WITT) cell lines, along with non-malignant human cholangiocytes (H69), were used. All cell lines were kindly provided by Prof. Alvaro (Department of Translational and Precision Medicine, Sapienza University of Rome, Rome, Italy). The CCLP1 cell line was cultured in RPMI 1640 medium containing 10% bovine fetal serum and 1% penicillin/streptomycin (Sigma-Aldrich, St. Louis, MO, USA). The hormonally integrated H69 medium described in a previous paper [33] was used to maintain the H69 and WITT cell lines.

Peripheral blood mononuclear cells (PBMCs) were isolated from the whole blood of anonymous healthy donors. Informed consent was obtained before sample collection. The study was conducted following the Declaration of Helsinki and approved by the local ethics committee (Italian Committee on Human Research, REF. TS/CEUR-CET/CEL n.20230044811—3 November 2023). PBMC isolation and culture were performed following previously described protocols [34].

Cells were maintained at 37 °C in a humidified atmosphere of 5% CO_2_. Human cell lines were periodically tested for mycoplasma by using the MycoAlert PLUS detection kit (Lonza, Walkersville, MD, USA).

### 2.6. Cell Viability Assay

CCLP1 (4 × 10^3^ cells/well), H69 (4 × 10^3^ cells/well) and PBMCs (8 × 10^3^ cells/well) were seeded in 96-well plates. The day after, cells were treated with increasing concentrations of PWE ranging from 1 to 256 μg/mL (1–2–4–8–16–32–64–128–256 μg/mL) for up to 72 h. The control group (0) received water equivalent to the maximum < 0.1% solvent used in the experimental settings. In CCLP1, cell viability was assessed at 24, 48, and 72 h of treatment, whereas in PBMCs and H69 cells only at 72 h. Cell viability was determined by CellTiter-Glo^®^ 2.0 Cell Viability Assay (Promega, Madison, WI, USA). At the end of the treatments, 100 μL of CellTiter-Glo^®^ 2.0 Reagent was added, and the contents were mixed for 2 min to ensure complete cell lysis. Luminescence was measured using a GloMax^®^ Discover Microplate Reader (Promega) after 10 min of incubation at room temperature.

### 2.7. Colony Formation Assay

CCLP1 cells were pre-treated with PWE (2–8–32–128 µg/mL) for 24, 48 and 72 h. Afterward, CCLP1 cells were seeded in 6-well plates at the density of 1 × 10^3^ cells/well with fresh medium and grown for 1 week to form colonies. Colonies were fixed with EtOH 95% for 30 min and stained with 1% crystal violet (Sigma-Aldrich) for 30 min. The plates were rinsed with water and left to dry at room temperature. Counting of clones was performed the following day.

### 2.8. Evaluation of Cell Viability in Tumor Spheroids

CCLP1 cells (1 × 10^4^ cells/well) were plated in 96-well Corning^®^ Costar^®^ Ultra-Low Attachment (ULA) round-bottomed plates (Corning Incorporated, Kennebunk, ME, USA) and incubated at 37 °C and 5% CO_2_ for 5 days to allow spheroid formation. Afterwards, 50 µL of culture medium was carefully replaced with fresh medium for control spheroids, or with fresh medium containing PWE at concentrations of 2–8–32–128 µg/mL. Images of the spheroids were captured 72 h after PWE treatment, and cell viability was assessed using CellTiter-Glo^®^ 3D Cell Viability Assay (Promega). Briefly, 100 μL of CellTiter-Glo^®^ 3D reagent was added to each well. The plate was shaken vigorously for 5 min to promote cell lysis, followed by a 25-min incubation at room temperature to allow stabilization of the luminescent signal. Luminescence was then measured by GloMax^®^ Discover Microplate Reader (Promega).

### 2.9. Wound Healing Assay

The evaluation of cell migration was performed by wound healing assay. CCLP1 cells were plated on a 24-well plate and grown to 90–95% confluency. A gap was generated using a sterile 200 μL pipette tip, and cells were allowed to migrate into the wound area up to 72 h in the presence or absence of PWE (2–8–32–128 µg/mL). The cells were monitored at 24-h intervals for 3 days, and photographs were collected at regular time intervals. Finally, the area of the wound closure was measured using ImageJ software (https://imagej.net/ij/index.html, NIH, accessed on 14 May 2025) and calculated by the following formula: (Area of original wound − Area of wound during healing)/Area of original wound.

### 2.10. Metabolic Profiling by Seahorse XF Assays

Seahorse Extracellular Flux XF Pro Analyzer (Agilent Technologies, Santa Clara, CA, USA) was used to measure oxygen consumption rate (OCR) and extracellular acidification rate (ECAR) by the means of the Seahorse XF Real-Time ATP Rate Assay (Agilent Technologies) protocol according to the manufacturer’s instructions. In detail, CCLP1 cells were seeded in a Seahorse XF Pro M (TC-treated) cell culture microplate at a density of 7 × 10^3^. The following day, cells were treated with 2, 8, or 32 µg/mL PWE and maintained in culture for an additional 24 h. A day before the experiment, the sensor cartridge was hydrated with 200 μL of calibrant solution and incubated overnight at 37 °C without CO_2_. On the day of the experiment, cells were washed once with preheated XF-DMEM medium (Agilent Technologies) supplemented with 1 mM pyruvate, 2 mM glutamine, and 10 mM glucose according to the protocol [35]. Subsequently, 180 μL of XF-DMEM medium was slowly added to each well, and cell cultures were allowed to equilibrate for 1 h at 37 °C in a no-CO_2_ incubator. Solutions of oligomycin and rotenone/antimycin A were made using XF-DMEM medium and placed into ports A and B of the sensor cartridge, respectively [35]. Seahorse XF Pro analysis was performed at 37 °C simultaneously measuring OCR (pmole O_2_/min) and ECAR (mpH/min). XF ATP Rate Assay measures OXPHOS and glycolysis’s contribution to ATP production. OCR and ECAR were measured before and after sequential injection of 1.5 µM oligomycin and 0.5 µM rotenone/antimycin A.

Data was derived from three independent experiments, where each sample was performed with at least 8 technical replicates. All data were assessed with Agilent Seahorse Analytics Software (https://seahorseanalytics.agilent.com, accessed on 27 March 2025) (Seahorse Bioscience, Agilent Technologies). Seahorse XF data were normalized to total protein content determined by post hoc Bradford quantification as previously described [35].

### 2.11. Quantitative Real-Time PCR (RT-qPCR)

Total RNA was extracted from 2 × 10^6^ cells using the RNeasy Plus Mini Kit (Qiagen, Hilden, Germany) following the manufacturer’s instructions. To synthesize complementary DNA (cDNA), 1 µg of total RNA was reverse transcribed using the iScript™ cDNA Synthesis Kit (Bio-Rad Laboratories, Hercules, CA, USA) on a GeneAmp 2720 Thermal Cycler (Applied Biosystems, Foster City, CA, USA) according to the following protocol: 25 °C for 5 min, 46 °C for 20 min, and 95 °C for 1 min. RT-qPCR experiments were performed in triplicate using TaqMan Gene Expression Assays (Thermo Fisher Scientific, Waltham, MA, USA) specific for human *GLUD1* (Hs03989560_s1, RefSeq NM_001318900.1) and *β-actin* (Hs01060665, RefSeq NM_001101.3) with the 7500 Fast Real-Time PCR system (Thermo Fisher Scientific). Relative gene expression levels were calculated using the 2^−ΔΔCt^ method, as previously reported [36].

### 2.12. Western Blot Analysis

CCLP1 cells (2 × 10^6^) were treated with PWE (2–8–32 μg/mL) or 20 μM IKK Inhibitor VII (IKK16, Sigma-Aldrich) for 24 h. Cells were lysed and protein separated by 12% sodium dodecyl sulfate-polyacrylamide gel electrophoresis (SDS-PAGE) and transferred onto nitrocellulose membranes as per the protocol described in [37]. The membranes were immunostained first overnight at 4° C with anti-GDH (PA5-69381, Thermo Fisher Scientific), anti-NF-κB/p65 (ab16502, Abcam, Cambridge, MA, USA), or anti-β–actin (ab8227, Abcam) primary antibodies, then at room temperature for 1 h with a goat anti-rabbit secondary antibody conjugated to horseradish peroxidase (HRP) (Santa Cruz Biotechnology, Santa Cruz, CA, USA). Protein bands were detected using the Chemidoc™ XRS detection system (Bio-Rad Laboratories). Image acquisition and densitometric analysis were performed using Image Lab software (version 6.1, Bio-Rad Laboratories).

### 2.13. In Silico Analysis of GLUD1 Promoter

The sequence of the *GLUD1* gene from *Homo sapiens* (Gene ID: 2746, NCBI Reference Sequence: NG_013010.1) was retrieved as a FASTA file from National Center for Biotechnology Information (NCBI) Genome Browser (https://www.ncbi.nlm.nih.gov/gene (accessed on 21 January 2025)) and used for the in silico analysis. The promoter region was selected as the 3 kb upstream sequence from the start codon. TRANSFAC database [38], integrated into the GeneXplain platform (www.genexplain.com), was employed to predict the potential binding sites of transcription factors within the *GLUD1* promoter region.

### 2.14. ChIP-qPCR

CCLP1 cells (8 × 10^6^), previously exposed to varying concentrations (2, 8, and 32 μg/mL) of PWE for 24 h, were cross-linked with 1% formaldehyde at 37 °C for 10 min to preserve protein-DNA interactions. The cells were lysed, and then the chromatin was fragmented by sonication as previously described in [37]. Chromatin immunoprecipitation (ChIP) was performed by incubating the samples overnight at 4 °C on a rocking platform with 5 μg of anti-NF-κB/p65 antibody (ab16502, Abcam) and 40 μL of A/G PLUS agarose beads (SC-2003, Santa Cruz Biotechnology). After reverse cross-linking with RNase and Protease K (Sigma-Aldrich), the samples were purified using DNA Gel Extraction Kit (Norgen Biotek Corp., Thorold, ON, Canada). Quantitative PCR (qPCR) analysis was carried out using PowerUp™ SYBR™ Green Master Mix (Thermo Fisher Scientific) and 20 pmol of gene-specific forward (CGAGATGGTGCCACTACAC) and reverse (AACAGAGGGAGACCCTATCTC) primers (Eurofins Genomics, Ebersberg, Germany) designed to amplify the 201 bp region of the human *GLUD1* promoter containing the NF-κB binding motif. The reactions were conducted on a 7500 Fast Real-Time PCR System (Thermo Fisher Scientific).

### 2.15. RNA Interference and Transient Transfection

The human *GLUD1* gene was transiently silenced by RNA interference. Briefly, CCLP1 cells were transfected for two consecutive days with a specific small interfering RNA (siRNA) targeting *GLUD1* (s13, Silencer^®^ Select Validated, Thermo Fisher Scientific) or with a scrambled control siRNA (4390843, Thermo Fisher Scientific) using Lipofectamine RNAiMAX Reagent (Thermo Fisher Scientific). Cell viability was measured 72 h after transfection, according to the protocol reported in Section 2.6.

For NF-κB activity assays, CCLP1 cells were transiently transfected with the NF-κB reporter plasmid pGL3–5 × NF-κB, containing a firefly luciferase gene under the control of five NF-κB response elements (5′-GGGACTTTCC-3′) upstream of a minimal TATA box promoter. Cells were co-transfected with pRL-CMV (Promega), expressing *Renilla* luciferase, to normalize transfection efficiency. After 24 h, cells were treated with PWE (2, 8, or 32 µg/mL). Twenty-four hours later, cells were lysed, and firefly and *Renilla* luciferase activities were measured using the Dual-Luciferase^®^ Reporter Assay System (Promega) according to the manufacturer’s instructions. Luminescence was quantified using a GloMax^®^ Discover Microplate Reader (Promega).

### 2.16. Measurement of hGDH Activity in Cell Extracts

GDH activity in cellular lysates from CCLP1 cells was evaluated with or without PWE (5–12.5–50–87.5–125–200–250 ng/mL). CCLP1 (1 × 10^7^ cells) were harvested and washed twice with ice-cold phosphate-buffered saline (PBS), then subjected to freeze-thaw lysis, as described in [37]. A total amount of 150 μg of the cellular extract was added to a 250 μL reaction mixture composed of 100 mM Tris-HCl (pH 7.4), 50 mM NH_4_Cl, 0.4 mM NADH, and 1.5 mM α-KG. The enzymatic activity was determined in 96-well plates by monitoring the decrease in NADH absorbance at 340 nm over 10 min using a Multiskan SkyHigh Microplate Reader (Thermo Fisher Scientific).

### 2.17. Inhibition of Purified Bovine Liver GDH Enzyme Activity

The enzymatic activity of purified GDH from bovine liver (Sigma-Aldrich, Type II with a specific activity: 40 U/mg protein) was measured spectrophotometrically by monitoring the consumption of NADH in a reductive amination reaction using ammonium chloride and α-KG as substrates, as previously described [23]. The reaction mixture included 0.3 U of purified GDH, 100 mM Tris-HCl pH 7.4, 50 mM NH_4_Cl, 0.4 mM NADH, and 1.5 mM α-KG in a total volume of 250 μL. Reactions were carried out in 96-well plates and were performed with and without PWE at different concentrations (0.625, 1.25, 2.5, 3.75, 5, 8.75, 12.5, 50, 125, 250, 500 ng/mL) and punicalagin, ellagic acid, quercetin and EGCG at final concentrations of 0.1, 0.2, 0.5, 1, 2, 5, and 10 µM. The consumption of NADH was recorded at 340 nm over 5 min using a Multiskan SkyHigh Microplate Reader (Thermo Fisher Scientific).

Furthermore, the Km (half-saturation constant) of α-KG for bovine GDH1 and the inhibition mechanism of punicalagin were determined by primary Lineweaver–Burk plots (namely double reciprocal plots). Finally, the secondary plot of the slopes (*Km*/*Vmax*) against inhibitor concentrations was used for calculating *K_i_* (the inhibition constant) for punicalagin. The *K_i_* obtained from the secondary plot was in agreement with the value obtained according to the equation for competitive inhibitors [39].

$$\frac{1}{V}=\frac{Km*\left(1+\frac{\left[I\right]}{Ki}\right)}{Vmax*[S]}+\frac{1}{Vmax}$$

### 2.18. Statistical Analysis

Statistical analyses were conducted using GraphPad Prism software, version 8.0.2 (La Jolla, CA, USA). Data are expressed as mean ± standard deviation (SD) from three independent experiments, each performed in at least triplicate. The Shapiro-Wilk test at an *α*-level of 0.05 was used to assess for data normality. One-way ANOVA was used to determine statistical significance, with Dunnett’s post hoc test applied when comparing treated groups to the unstimulated control. Statistical significance is represented in the figures by asterisks: * *p* < 0.05; ** *p* < 0.01; *** *p* < 0.001.

## 3. Results

### 3.1. Quali-Quantitative Profiling of Phenolic Compounds in PWE

The qualitative and quantitative analysis of phenolic compounds, including punicalagin, flavonoids, and phenolic acids in PWE, was carried out by LC-MS/MS. Metabolite identification was achieved through comparison with reference standards and through sMRM, enabling accurate separation and quantification of compounds.

As shown in Table 1, punicalagin was the predominant phenolic compound, detected at a concentration of 31.72 ± 2.59 mg/g, consistent with the polyphenol profile reported in juice and peel of *P. granatum* L. varieties [40]. Ellagic acid dihydrate was detected at a concentration of 1.18 ± 0.03 mg/g. Gallic acid was also identified at 0.08 ± 0.004 mg/g. Among the flavonoids, (-)-Epigallocatechin gallate hydrate exhibited the highest concentration at 0.19 ± 0.005 mg/g, followed by phloridzin (0.05 ± 0.005 mg/g) and baicalein/apigenin (0.04 ± 0.005 mg/g each). Other flavonoids (baicalin and rutin hydrate) were found in trace amounts. The complete flavonoid and phenolic acids panel investigated in PWE is reported in Supplementary Table S1.

### 3.2. PWE Selectively Reduces the Viability and Long-Term Proliferation of CCLP1 Cells

**Figure 1:**
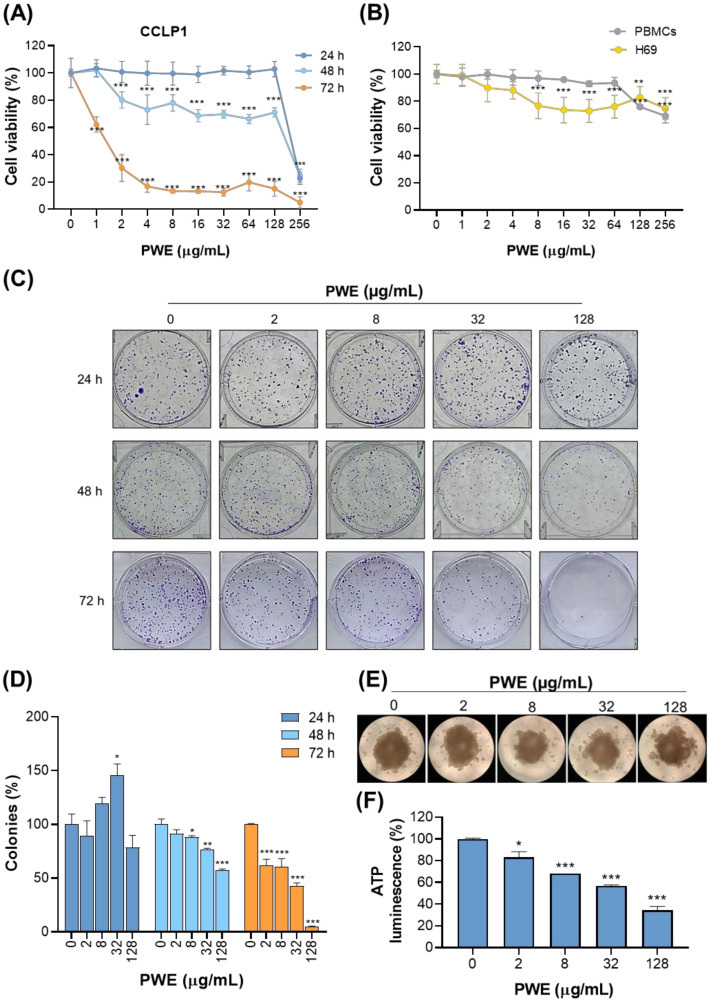
PWE inhibits the viability and clonogenic growth of CCLP1 cells. (**A**) CCLP1 cells were treated with increasing concentrations of PWE (0–256 μg/mL) and cell viability was assessed at 24–48–72 h. (**B**) Non-malignant H69 cholangiocytes and PBMCs from healthy donors were treated with the same PWE concentrations, and cell viability was measured after 72 h. (**A**,**B**) show cell viability normalized to untreated control cells (0) at each time point of the assay, with the viability of untreated cells set to 100%. (**C**,**D**) CCLP1 cells were exposed to PWE (2, 8, 32, 128 μg/mL) for 24, 48, and 72 h, followed by clonogenic assays. (**C**) Representative images of colony formation in Petri dishes are shown. (**D**) Quantification of colony numbers. (**E**) Representative images of 3D tumor spheroids treated with increasing concentrations of PWE for up to 72 h. (**F**) Quantification of cell viability in 3D tumor spheroids after 72 h treatment with PWE. In (**A**,**B**,**D**,**F**) data represent the mean ± SD of three independent experiments. Statistical analysis was performed using one-way ANOVA followed by Dunnett’s multiple comparison test (* *p* < 0.05; ** *p* < 0.01; *** *p* < 0.001).

Given the aggressive nature of iCCA and the scarcity of effective therapeutic options, we investigated the anticancer potential of PWE in CCLP1 cells by assessing its impact on cell viability and long-term proliferation. A dose-response analysis (0–256 μg/mL) over 24, 48, and 72 h revealed that PWE significantly reduced cell viability in a time- and dose-dependent manner in CCLP1 cells (Figure 1A). At 24 h, cell viability was unaffected up to 128 μg/mL, but declined markedly at 256 μg/mL, with only ~20% of cells remaining viable. At 48 h, the cytotoxic effect was more pronounced, with significant reductions (20–30%) observed at concentrations ranging from 2 to 128 μg/mL to reach ~80% at 256 μg/mL. At 72 h, even the lowest concentration of 1 μg/mL resulted in approximately 40% reduction in viability, with survival continuing to decline across higher doses and plateauing at ~15% between 4 and 128 μg/mL. At the highest concentration (256 μg/mL), cell viability was nearly abolished (Figure 1A).

To assess the selectivity of PWE, PBMCs from healthy donors and human non-malignant cholangiocytes (H69) were treated with the same concentration range of PWE for 72 h. In PBMCs, cell viability remained relatively stable across all concentrations, with only modest reductions (~30%) at the highest doses of 128 and 256 μg/mL (Figure 1B). In H69 cells, slight decreases in cell viability of about 25% were observed at concentrations above 8 μg/mL. These findings suggest that PWE exerts selective cytotoxic effects on malignant cells while sparing normal cells (Figure 1B)

We further explored the long-term antiproliferative effect of PWE through the colony formation assay. Cells were exposed to increasing concentrations of PWE (2–8–32–128 µg/mL) for 24, 48, 72 h and allowed to form colonies. A marked, dose- and time-dependent decrease in colony formation was observed, with nearly complete inhibition at the highest concentrations tested, indicating long-term growth suppression by PWE. While no significant effect was observed at 24 h, PWE reduced the number of colonies at 48 and 72 h (Figure 1C,D). Forty-eight-hour exposure caused a significant dose-dependent decrease in cell replication capacity, with a peak at 128 µg/mL, around 40% of that of the untreated control (Figure 1C,D). After 72 h, the colony-forming capacity was reduced by approximately 40–60% at low doses, while the highest concentration tested (128 µg/mL) caused almost complete inhibition (Figure 1C,D).

To evaluate the efficacy of PWE in a more physiologically relevant model, we assessed its effect on 3D tumor spheroids. Spheroids were treated with increasing doses of PWE up to 72 h. Consistent with the results obtained in 2D cell culture, a dose-dependent decrease in spheroid viability was observed, with substantial reductions at concentrations ≥32 μg/mL and ~70% loss at 128 μg/mL (Figure 1E,F).

Taken together, these findings provide compelling evidence that PWE selectively impairs CCLP1 cell viability, suppresses clonogenic potential, and exerts potent cytotoxic effects in 3D tumor spheroids, highlighting its potential as a selective therapeutic agent for CCA.

### 3.3. PWE Reduces Wound Closure in CCLP1 Cells

**Figure 2:**
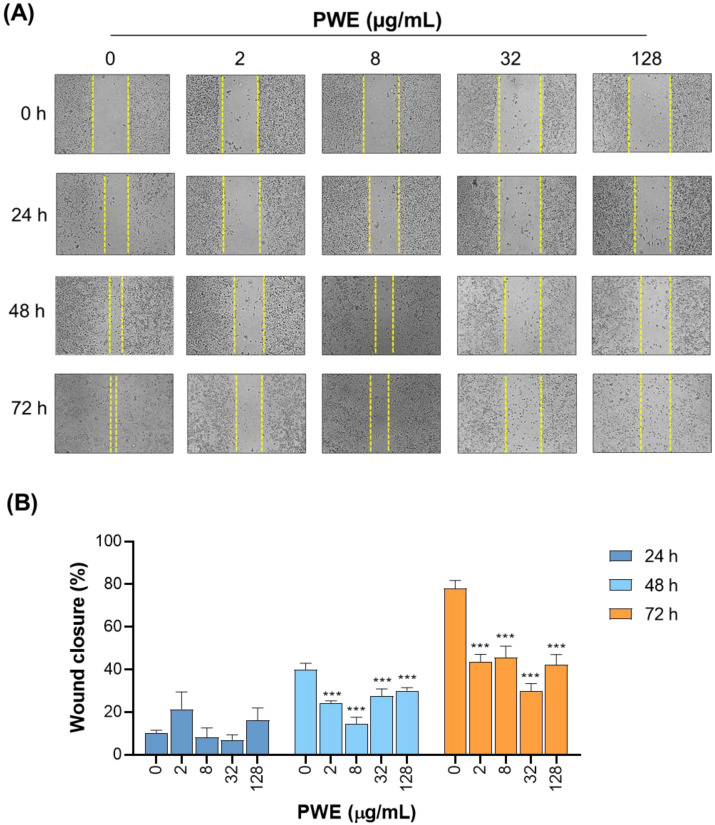
PWE inhibits CCLP1 cell migration. Wound healing assays were performed to assess the effect of PWE on the migratory capacity of CCLP1 cells. (**A**) Representative images of scratch closure at 0, 24, 48, and 72 h post-wounding in cells treated with increasing concentrations of PWE (2, 8, 32, and 128 μg/mL) or untreated controls (0 μg/mL). Yellow dashed lines mark the wound edges at each time point. (**B**) Quantification of wound closure over time expressed as the percentage of gap closure relative to the initial scratch width (0 h). Data represent the mean ± SD of three independent experiments. Statistical significance was determined using one-way ANOVA followed by Dunnett’s multiple comparison test (*** *p* < 0.001).

Next, we investigated the effect of PWE treatment on healing wounds, which reflects the migratory and motile capacity of cancer cells. CCLP1 cells were exposed to increasing concentrations of PWE (2–8–32–128 µg/mL), and wound closure was monitored at 24, 48, and 72 h. Figure 2 shows that the untreated cells had a normal ability to migrate, and the wound was almost completely healed within 72 h, while the PWE-treated groups showed a reduction in wound closure at 48 and 72 h (Figure 2A,B). After 72 h of treatment, a significant reduction in wound closure was observed at all tested concentrations. In particular, the concentration of 32 μg/mL proved to be the most effective at all times tested, reducing wound closure by approximately 50% compared to a control group after 72 h from treatment (Figure 2A,B). These results indicate that PWE treatment reduces wound closure in CCLP1 cells under the tested conditions. However, this reduction may not exclusively reflect an inhibition of cell migration, as the concentrations used in this assay are associated with decreased cell viability. Therefore, the reduced wound closure could also result from decreased cell proliferation and/or increased cell death rather than a direct anti-migratory effect.

### 3.4. PWE Reduces ATP Production and Extracellular Acidification in CCLP1 Cells

Given the phenotypic effects of PWE on CCLP1 cell proliferation and migration, we wondered whether PWE also affected cellular metabolism. To this end, we employed the Seahorse Extracellular Flux XF Pro Analyzer, which enables real-time monitoring of energy metabolism in live cells to measure ATP production rate, oxygen consumption rate (OCR), an indirect measure of mitochondrial respiration (OXPHOS), and the extracellular acidification rate (ECAR), an indirect measure of glycolytic activity.

**Figure 3:**
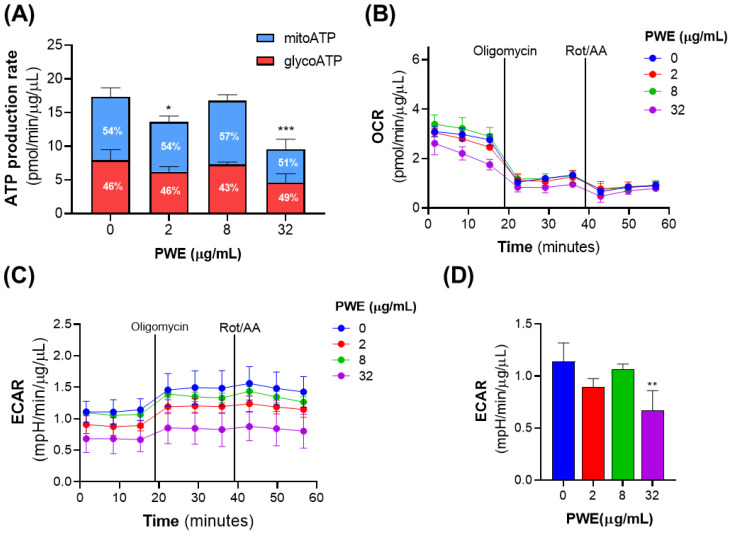
PWE lowers ATP levels and extracellular acidification in CCLP1 cells. Metabolic flux analysis was performed using the Seahorse XFe96 analyzer. Cells were treated with PWE (2, 8, 32 μg/mL) or left untreated (0) for 24 h. (**A**) Total intracellular ATP production, showing contributions from glycolysis (glycoATP, red) and oxidative phosphorylation (mitoATP, blue); relative percentages are indicated within each bar. (**B**) Oxygen consumption rate (OCR), and (**C**) extracellular acidification rate (ECAR), measured at baseline and following sequential addition of oligomycin and antimycin A/rotenone (Rot/AA). (**D**) Basal ECAR values extracted from (**C**) and compared across treatment groups. Data represent the mean ± SD of three independent experiments. Statistical significance was determined using one-way ANOVA followed by Dunnett’s multiple comparison test (* *p* < 0.05; ** *p* < 0.01; *** *p* < 0.001).

Analysis of ATP production revealed that PWE at doses of 2 and 32 μg/mL diminished the overall ATP production rate at ~20% and ~45%, respectively, of the control group. The percentages of glycolytic ATP (glycoATP) and mitochondrial ATP (mitoATP) remained largely unchanged across all conditions. Treatment with 8 µg/mL PWE did not affect total, glycoATP, or mitoATP production rates (Figure 3A).

OCR seems not to be affected by treatment with 2 and 8 µg/mL PWE; in fact, basal OCR was the same the untreated cells. Treatment with 32 µg/mL PWE resulted in a ~40% significant decrease in basal OCR (*p* < 0.001, Tukey’s test). Furthermore, proton leak and non-mitochondrial respiration remained unchanged across all treatment conditions (Figure 3B).

Under basal conditions, ECAR was significantly decreased (~30%) in cells treated with 32 µg/mL of PWE compared to untreated cells, and a slight but not significant decrease was observed in cells treated with 2 µg/mL of PWE. No changes were detected at 8 µg/mL (Figure 3C,D). Due to the inhibition of mitochondrial ATPase caused by the addition of oligomycin, an increase in ECAR was observed in both treated and untreated cells, as expected (Figure 3C).

In conclusion, PWE induces measurable metabolic effects primarily at the highest tested concentration (32 μg/mL), with reduced total ATP production, decreased basal OCR, and lower extracellular acidification. Effects at lower concentrations are minimal or not statistically significant, consistent with an impairment of cancer cell bioenergetics at higher doses.

### 3.5. PWE Reduces GDH Expression via NF-κB Signaling in CCLP1 Cells

The results presented above suggest that PWE impairs CCLP1 cell growth and invasive potential, likely through metabolic alterations that lead to a reduction in total ATP production and extracellular acidification. Since glutaminolysis is a relevant pathway to ensure nutrient sources in CCA cancer cells, we next investigated whether PWE affects this metabolic pathway, as previously demonstrated in hepatocellular carcinoma [21].

**Figure 4:**
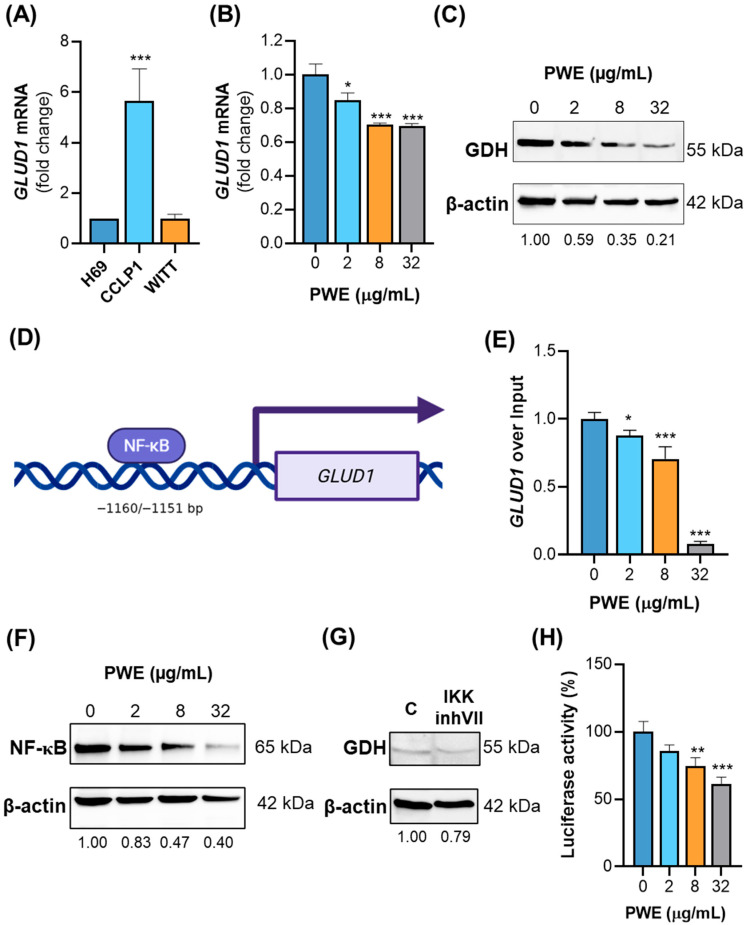
PWE reduces hGDH1 expression through NF-κB signaling. (**A**) *GLUD1* mRNA expression levels were measured by real-time PCR in H69 CCLP1 and WITT CCA cell lines. (**B**,**C**) CCLP1 cells were treated with PWE (2, 8, and 32 μg/mL) or left untreated (0), and *GLUD1* mRNA (**B**) and hGDH1 protein levels (**C**) were evaluated by real-time PCR and Western blotting, respectively. (**D**) Schematic representation of the human *GLUD1* gene promoter region showing a predicted NF-κB binding site location. (**E**) CCLP1 cells were treated with PWE (2, 8, and 32 μg/mL), and a chromatin immunoprecipitation (ChIP) analysis was performed using an antibody against NF-κB/p65. Following immunoprecipitation, the *GLUD1* promoter region was analyzed by real-time PCR. Fold changes in promoter occupancy were calculated relative to untreated cells (0 μg/mL). (**F**) Western blot analysis of total NF-κB (p65) protein levels in CCLP1 cells treated as in (**E**). (**G**) CCLP1 cells treated with or without IKK inhibitor VII (IKKinhVII) were analyzed by Western blot using a GDH-specific antibody. (**H**) NF-κB-dependent luciferase activity was measured in CCLP1 cells transiently transfected with the pGL3–NF-κB reporter plasmid and treated with PWE. In (**C**,**F**,**G**) protein levels were normalized to β-actin. In (**A**,**B**,**E**,**H**) data are expressed as means ± SD from three experiments. Statistical significance was determined using one-way ANOVA followed by Dunnett’s multiple comparison test (* *p* < 0.05; ** *p* < 0.01; *** *p* < 0.001).

Firstly, we analyzed *GLUD1* gene expression levels across non-malignant (H69) and tumoral cholangiocytes from iCCA (CCLP1) and eCCA (WITT). We found that *GLUD1* mRNA was more expressed in CCLP1 cells than in H69 and WITT cells (Figure 4A), supporting a potential role for hGDH1 in the metabolic phenotype of CCLP1 cells.

Then, we assessed whether PWE affects the GDH expression levels by measuring gene and protein expression in CCLP1 cells treated with or without PWE. Real-time PCR and Western blot analyses revealed a dose-dependent decrease in *GLUD1* mRNA and hGDH1 protein levels following treatment of CCLP1 cells with PWE (2–8–32 µg/mL) (Figure 4B,C). Specifically, *GLUD1* mRNA levels were reduced by approximately 15% at the lowest concentration (2 µg/mL) and by 30% at the higher concentrations of PWE (8 and 32 µg/mL) (Figure 4B). The downregulation of hGDH1 protein was even more pronounced, with dose-dependent reductions ranging from 40% to 80% (Figure 4C). To address the potential causal role of GDH inhibition in the anti-proliferative effects of PWE, *GLUD1* was transiently silenced in CCLP1 cells. *GLUD1* knockdown significantly reduced cell proliferation, suggesting that GDH downregulation contributes, at least in part, to the anti-tumor activity of PWE (Figure S3).

The reduction in hGDH1 expression following PWE treatment suggested that PWE might also exert transcriptional regulation of the *GLUD1* gene. Using the TRANSFAC databases and software version 2.0 2025.1 licensed from geneXplain, we performed an in silico analysis of the human *GLUD1* promoter region, which revealed a proximal, putative NF-κB binding site located at position −1160/−1151 bp upstream of the translation start site (Figure 4D). Given the established role of the p50/p65 NF-κB heterodimer in gene regulation, we assessed NF-κB activity at this site by ChIP experiments with a p65-specific antibody and primers spanning a 201 bp region encompassing the predicted binding site. We observed a dose-dependent reduction in p65 occupancy at the NF-κB binding site following PWE treatment, indicating suppressed transcriptional activation (Figure 4E). Notably, at a concentration of 32 μg/mL, NF-κB binding to these transcription factor binding sites (TFBS) was completely abolished. In parallel, we observed a dose-dependent decrease in total p65 expression following PWE treatment (Figure 4F). To confirm the involvement of NF-κB signaling in *GLUD1* regulation, cells were treated with the IKK inhibitor VII, which blocks NF-κB activation. This inhibition also reduced hGDH1 protein levels, reinforcing the role of NF-κB in *GLUD1* transcription (Figure 4G). Consistently, PWE treatment reduced NF-κB transcriptional activity in a luciferase reporter assay. Notably, PWE at 32 μg/mL decreased NF-κB transactivation by approximately 60% compared with untreated cells (Figure 4H).

Collectively, our data suggest that PWE downregulates *GLUD1* transcription by suppressing NF-κB signaling, thereby reducing hGDH1 expression and contributing to metabolic alterations in CCLP1 cells.

### 3.6. PWE Inhibits GDH Activity

**Figure 5:**
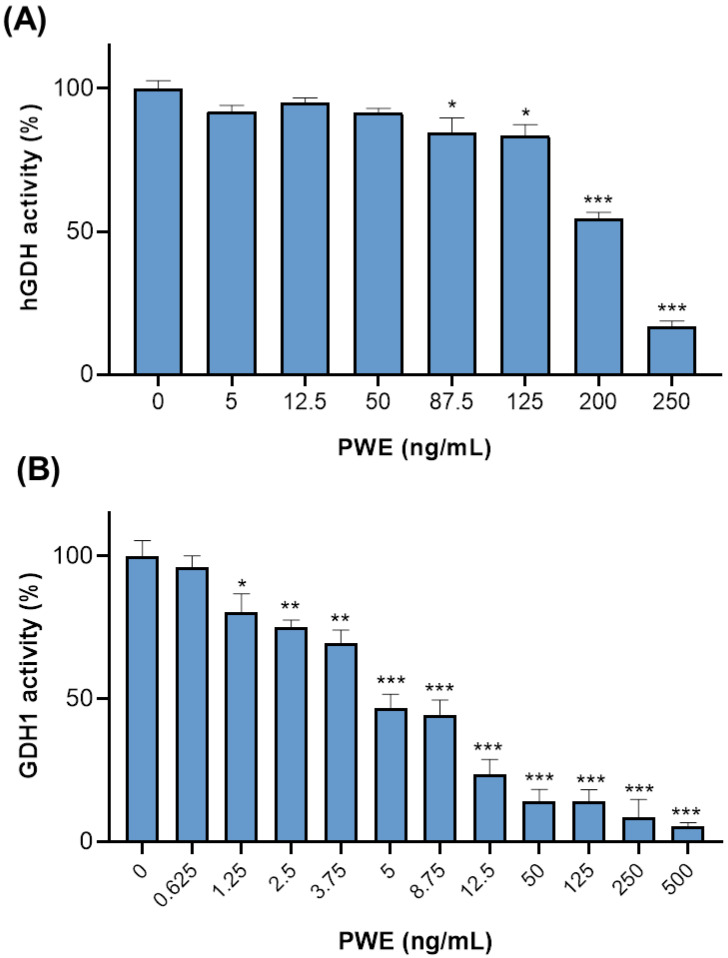
PWE inhibits GDH activity. (**A**) hGDH activity in CCLP1 cell lysates was assessed following treatment with α-KG alone (0) or in combination with PWE (2, 8, and 32 μg/mL). (**B**) Effect of PWE on the activity of purified bovine liver GDH1 (GDH1). Enzymatic activity was initiated by the addition of α-KG in the absence (0) or presence of increasing concentrations of PWE. The enzymatic activity was monitored by measuring the decrease in NADH absorbance at 340 nm over 10 min. In (**A**,**B**) residual activity (%) is shown as the mean ± SD from at least three independent experiments. Statistical significance was determined using one-way ANOVA followed by Dunnett’s multiple comparison test (* *p* < 0.05; ** *p* < 0.01; *** *p* < 0.001).

Having established that PWE affects hGDH1 expression, playing a relevant role in CCA cells, we evaluated the direct effect of PWE on GDH activity. Firstly, we assessed whether PWE could inhibit endogenous hGDH activity in CCLP1 cells. Results show a concentration-dependent inhibition of hGDH activity in CCLP1 cell extracts treated with different concentrations of PWE (5–250 ng/mL), with reductions of 54 ± 2.2% and 16.9 ± 1.8% at 200 and 250 ng/mL, respectively, compared to untreated controls (Figure 5A).

Next, we measured the activity of purified GDH1 from bovine liver, which shows an amino acid sequence similarity of about 98% with hGDH1 and is commonly used as a biochemical model of the human enzyme. Nevertheless, potential species-specific differences should be considered when extrapolating inhibition kinetics. Interestingly, by adding PWE within the range of 0.625–500 ng/mL, we observed an inhibition of bovine GDH1 activity in a concentration-dependent manner, and 50% inhibition in the presence of PWE between 5 and 8.75 ng/mL (Figure 5B).

### 3.7. Punicalagin Is a New Inhibitor of GDH

Based on the effect on GDH activity and on the polyphenol profile of PWE, we investigated the inhibition of purified bovine liver GDH1 activity by punicalagin and ellagic acid, the main compounds present in PWE. EGCG and quercetin were used as known inhibitors of GDH1 activity [23].

**Figure 6:**
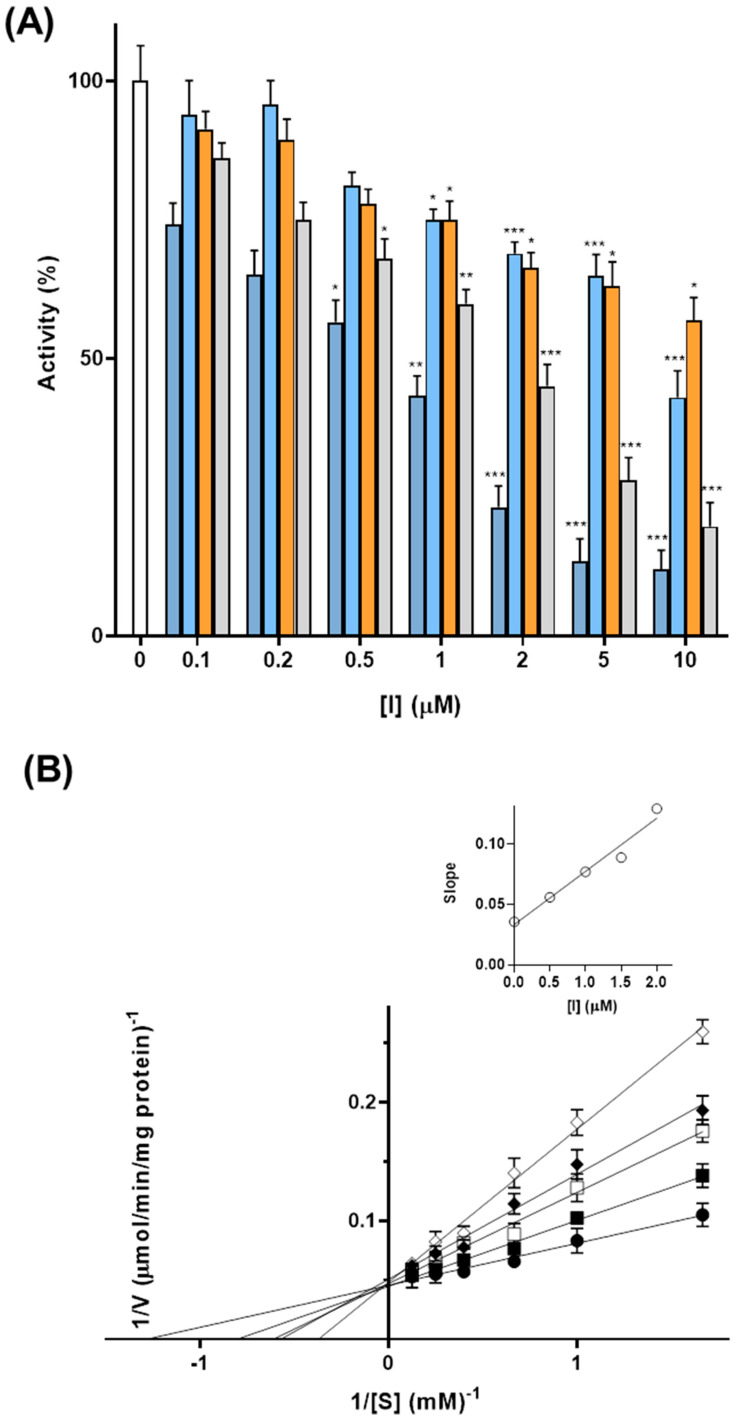
Inhibitors of the activity of purified bovine liver GDH1. (**A**) The GDH1 activity was initiated by adding NH_4_Cl in the absence (white column) or in the presence of increasing external concentrations of Punicalagin (blue columns), Ellagic acid (light blue columns), Quercetin (orange columns) or EGCG (light grey columns). The enzymatic activity was monitored by measuring the decrease in NADH absorbance at 340 nm over 10 min. The residual activities (%) in presence of Punicalagin, Ellagic Acid, and EGCG are the means ± SD of at least three independent experiments. Statistical significance was determined using one-way ANOVA followed by Dunnett’s multiple comparison test (* *p* < 0.05; ** *p* < 0.01; *** *p* < 0.001). (**B**) Lineweaver–Burk plot reporting the GDH1 activity at the indicated concentrations of α-KG in the absence (●) or in the presence of 0.5 (■), 1 (□), 1.5 (⬥), and 2 (◇) μM of punicalagin. The values shown are the means ± SD for three independent measurements. The insert shows the slope of Lineaweaver–Burk plots versus [I] used for determining the inhibition constant Ki.

Punicalagin inhibited GDH1 activity in a concentration-dependent manner in a range of 0.1–10 μM (Figure 6A). A residual activity of about 50% was observed in the presence of a concentration between 0.5–1 μM. In contrast, ellagic acid showed a minor inhibitory effect in the same range, with a residual activity of about 50% in the presence of 10 μM, as well as quercetin, in good agreement with previous data [23]. Interestingly EGCG, a potent inhibitor of GDH, showed a residual activity of about 50% at a concentration of 2 μM.

Considering the major effect of punicalagin compared to ellagic acid, we investigated its inhibition mechanism by double-reciprocal plots from the reciprocal of initial rates versus the reciprocal of α-KG concentrations in the presence of a fixed saturating concentration of ammonia and NADH and with or without different concentrations of punicalagin (Figure 6B)**.** The Michaelis–Menten (half-saturation) constant (Km) of the purified bovine liver GDH1 was 0.78 ± 0.04 mM, and the Vmax was 22.00 ± 1.75 μmol/min/mg protein for α-KG, in good agreement with the previously measured values [23].

Interestingly, punicalagin is a competitive inhibitor for α-KG; in fact, the Vmax values are the same, whereas Km_app_ values increase in the presence of different tested concentrations of punicalagin (0.2, 0.5, 1, 1.5 and 2 μM) (Figure 6B)**.**

By the secondary plot of slope (K_m_/V_max_) against the tested concentrations, used to determine the inhibitor constant, the Ki for punicalagin is 0.75 μM for purified liver bovine GDH1.

## 4. Discussion

Cholangiocarcinoma (CCA) is a rare but highly aggressive cancer, with increasing global incidence and mortality driven by the rising epidemiological trend of iCCA [1]. The poor prognosis is largely due to the asymptomatic early stages, often leading to late diagnosis and limited treatment effectiveness [2]. In this context, the identification of novel molecular vulnerabilities is urgently needed.

Metabolic alterations are key features of cancer cells and play a particularly relevant role in iCCA. New insights into the “metabolic life” of different tumor cells have led to novel therapeutic strategies and the evaluation of possible treatments. In iCCA, metabolic reprogramming includes enhanced glycolysis and OXPHOS, both essential for ATP production and biosynthesis of macromolecules [41]. Moreover, recent evidence highlights that mutations in metabolic enzymes occur in iCCA, specifically IDH, leading to the accumulation of the oncometabolite 2-hydroxyglutarate (2-HG) and consequent epigenetic alterations [42]. Alterations in other signaling pathways, including the fibroblast growth factor receptor 2 (FGFR2) [43], have further supported the development of targeted therapeutic strategies. Additionally, modulation of metabolic enzymes, such as those involved in lipid metabolism, represents another hallmark of iCCA [44]. Starting from these observations, the identification of new targets and potential molecules or compounds that can modulate these dysregulated metabolic pathways presents a promising avenue for treatment.

Within this framework, we explored the potential of pomegranate waste extract (PWE) on CCLP1 cells as a model of iCCA. In recent years, pomegranate has shown many different effects, among which are anticancer effects with demonstrated efficacy in many types of tumors, including breast, lung, colon, and liver cancers [45], antioxidant, and anti-inflammatory properties. Interestingly, pomegranate intake has shown a beneficial effect on liver metabolic function in metabolic disorders, suggesting a potential interaction with hepatic metabolic pathways [46]. Therefore, we hypothesized that PWE could interfere with metabolic reprogramming in iCCA cells.

In our experiments, we found that PWE selectively reduces CCLP1 cell viability while having no effect on non-tumoral cells, including PBMCs and non-malignant human cholangiocytes (H69) (Figure 1B). PWE significantly lowers ATP production, basal oxygen consumption rate (OCR), and extracellular acidification rate (ECAR) primarily at the highest tested concentration, indicating an impairment of cancer cell bioenergetics that involves both mitochondrial respiration and glycolytic activity. Although effects on non-tumoral cells were minimal at the tested concentrations, further studies are warranted to confirm selectivity across a broader panel of non-malignant hepatic cells.

To further elucidate the underlying mechanism, we examined whether PWE could modulate glutaminolysis, a key metabolic pathway altered in iCCA. GLS1, a component of the glutaminolysis, is overexpressed in iCCA [21]. However, little is known about the downstream mitochondrial hGDH1 that catalyzes the reversible conversion of glutamate to α-KG and ammonia, linking amino acid metabolism to the TCA cycle [47]. Due to its kinetic properties [48], the oxidative deamination of hGDH is preferred under physiological conditions, especially in tissues with low ammonia levels, thereby supporting TCA cycle anaplerosis and ATP production. hGDH1 was found overexpressed in HCC cells [23] and in eCCA [22], suggesting its involvement in liver tumor metabolism.

In line with these observations, we show that *GLUD1*, the gene encoding hGDH1, is overexpressed in CCLP1 cells, and we demonstrate that PWE downregulates *GLUD1* gene expression through dose-dependent reductions in NF-κB (p65). This effect may be mediated by punicalagin or ellagic acid, major components of PWE, which are known to inhibit NF-κB pathway in tumor cells [49,50]. Punicalagin suppresses IκBα phosphorylation and reduces nuclear translocation of p65/p50 subunits, attenuating inflammatory responses [51,52]. This finding suggests a long-term regulatory mechanism at the transcriptional level. Although the present study supports an NF-κB-dependent regulation of *GLUD1*, further studies evaluating NF-κB nuclear translocation would help to better define the molecular mechanisms underlying the inhibitory effects of PWE.

Beyond transcriptional regulation, we demonstrated that PWE inhibits the activity of both bovine purified GDH1 and hGDH in CCLP1 cell extract at very low concentrations. By reducing GDH activity, the conversion of glutamate to α-KG is limited, impairing the TCA cycle and ATP production, potentially driving iCCA cells toward metabolic exhaustion and reduced viability.

We further demonstrate that punicalagin acts as a novel competitive inhibitor of GDH, with a low Ki (~0.75 μM), highlighting its strong inhibitory potential comparable to known inhibitors [23,26]. Given the central role of GDH in glutamine metabolism, this finding provides mechanistic support for the metabolic effects observed in intact cells.

Beyond the therapeutic implications, the valorization of PWE aligns with the circular economic principle. Pomegranate juice production generates substantial inedible by-products (peels and seeds, ~54% of fruit weight), totaling ~1.62 million tons annually [53]. Developing bioactive extracts from this biomass represents a sustainable strategy combining environmental and biomedical benefits.

Although the present study demonstrates that PWE modulates GDH expression and activity in vitro, the translational and therapeutic relevance of these findings should be interpreted considering the limited systemic bioavailability of punicalagin. Reported plasma levels of intact punicalagin after oral intake are generally very low, suggesting that the systemic achievable in vivo concentrations may be lower than those tested in vitro.

However, in our study, we also observed that ellagic acid, a hydrolysis product and bioavailable metabolite of punicalagin [54], also modulates GDH activity, potentially supporting GDH-targeting effects in vivo. Therefore, although intact punicalagin exposure may be limited systemically, its metabolic conversion to ellagic acid could partially mediate GDH modulation under physiologically achievable conditions. Further in vivo studies are essential to validate efficacy under physiologically relevant exposure and to clarify the contribution of punicalagin and its metabolites, ultimately informing potential dietary or therapeutic applications.

## 5. Conclusions

In conclusion, our study demonstrates that PWE impairs iCCA cell proliferation and growth by targeting glutamine metabolism through GDH1 inhibition at both enzymatic and transcriptional levels. Mechanistically, PWE reduces *GLUD1* expression via suppression of NF-κB signaling and directly inhibits GDH activity, thereby limiting the conversion of glutamate to α-ketoglutarate and affecting TCA cycle anaplerosis and cellular bioenergetics. Among the bioactive compounds identified in PWE, punicalagin acts as a competitive inhibitor of GDH, providing mechanistic support for the metabolic effects observed in CCLP1 cells.

Overall, these findings highlight GDH-dependent glutamine metabolism as a potential metabolic vulnerability in iCCA and support the therapeutic potential of GDH inhibition. Moreover, punicalagin may represent a valuable lead molecule for the development of metabolism-targeted adjunctive or preventive strategies.

## Figures and Tables

**Table 1:** Phenolic compounds found in pomegranate extract (PWE). Values are expressed as mean ± standard deviation.

Phenolic Compound	mg/g
Punicalagin	31.72 ± 2.59
Ellagic acid dihydrate	1.18 ± 0.03
Gallic acid	0.08 ± 0.004
(-)-Epigallocatechin gallate hydrate	0.19 ± 0.005
Phloridzin	0.05 ± 0.005
Baicalein	0.04 ± 0.005
Apigenin	0.04 ± 0.005
Rutin hydrate	0.02 ± 0.005
Baicalin	0.01 ± 0.005

## Data Availability

The data used to support the findings of this study are available from the corresponding authors upon request.

## Supplementary Materials

The following supporting information can be downloaded at: https://www.mdpi.com/article/10.3390/biom16030449/s1, Table S1. Optimized Q1 mass, product ion and parameters for sMRM experiment of phenolic compounds. Table S2. Linearity and sensitivity data. Figure S1. Extract Ion Chromatogram (XIC) of Multi Reaction Monitoring (MRM) of the optimized 43 phenolic acids and punicalagin. Figure S2. Extract Ion Chromatogram (XIC) of Multi Reaction Monitoring (MRM) of the optimized 38 flavonoids. Figure S3. *GLUD1* knockdown reduces CCLP1 cell viability. Original images of Western Blotting can be found in Supplementary Materials.
