# Supplementary material for: Inhibition of Glutamate Dehydrogenase as a Potential Strategy to Modulate Intrahepatic Cholangiocarcinoma Cell Metabolism
Source: Biomolecules. 2026 Mar 17;16(3):449. doi: 10.3390/biom16030449 (PMC13023441; doi:10.3390/biom16030449)
Supplement: Supplementary file 1 [file biomolecules-16-00449-s001.zip › biomolecules-4177390-supplementary.pdf]

Table S1. Optimized Q1 mass, product ion and parameters for sMRM experiment of Phenolic Compounds.

Metabolite	Metabolite Class	Precursor ion (*m/z*)	Product ion (*m/z*)	DP[a]	EP[b]	CE[c]	CXP[d]	RT[e](min)
Quinaldic Acid	Internal Standard	174.05 $[M+H]^+$	128.0	70	10	30	9	2.57
punicalagin anomer	Phenolic compound	541.6 $[M-2H]^{2-}$	601.4	-70	-13	-38	-23	2.38
punicalagin anomer b	Phenolic compound							3.72
o-Anisic Acid	Phenolic Acids	152.8 $[M+H]^+$	135.1	70	10	17	20	4.78
2,3-Dihydroxybenzoic acid	Phenolic Acids	153.0 $[M-H]^-$	109.0	-49	-10.9	-19	-5	2.94
Chlorogenic acid	Phenolic Acids	353.1 $[M-H]^-$	190.8	-49	-10.9	-20	-10	3.99
Syringic acid	Phenolic Acids	197.0 $[M-H]^-$	152.9	-49	-10.9	-16	-16	4.17
p-Coumaric acid	Phenolic Acids	163.0 $[M-H]^-$	119.1	-49	-10.9	-12	-10	4.72
m-Hydrocoumaric acid	Phenolic Acids	165.0 $[M-H]^-$	120.6	-49	-10.9	-18	-7	4.78
Ferulic acid	Phenolic Acids	193.1 $[M-H]^-$	134.1	-49	-10.9	-20	-4	5.62
Sinapic acid	Phenolic Acids	223.1 $[M-H]^-$	163.5	-49	-10.9	-19	-16	6.01
Aspirin	Phenolic Acids	179.1 $[M-H]^-$	136.7	-49	-10.9	-12	-9	5.72
trans-Cinnamic acid	Phenolic Acids	147.0 $[M-H]^-$	103.0	-49	-10.9	-14	-10	7.66
4-Hydroxybenzoic acid	Phenolic Acids	137.0 $[M-H]^-$	92.7	-49	-10.9	-18	-7	1.84
2,6-Dihydroxybenzoic acid	Phenolic Acids	153.0 $[M-H]^-$	108.7	-49	-10.9	-18	-19	6.44
Dihydrocaffeic acid	Phenolic Acids	181.1 $[M-H]^-$	162.8	-49	-10.9	-19	-15	7.91
Caffeic acid	Phenolic Acids	179.0 $[M-H]^-$	134.4	-49	-10.9	-20	-7.5	3.17
Phloretic acid	Phenolic Acids	165.1 $[M-H]^-$	148.7	-49	-10.9	-20	-9	8.67
Hydroferulic acid	Phenolic Acids	195.1 $[M-H]^-$	135.7	-49	-10.9	-15	-5.6	4.9
Ellagic acid dihydrate	Phenolic Acids	301.0 $[M-H]^-$	144.8	-49	-10.9	-43	-17	7.1
5-Methoxysalicylic acid	Phenolic Acids	167.0 $[M-H]^-$	107.6	-49	-10.9	-30	-14	6.6
Catechol	Phenolic Acids	109.0 $[M-H]^-$	108.9	-49	-10.9	-11	-31	1.1
Gentisic acid	Phenolic Acids	153.0 $[M-H]^-$	107.7	-49	-10.9	-27	-7	2.19
4-Acetocatechol	Phenolic Acids	151.0 $[M-H]^-$	107.3	-49	-10.9	-27	-9	2.64
4-Methylcatechol	Phenolic Acids	123.0 $[M-H]^-$	94.5	-49	-10.9	-22	-14	2.9
2,6-Dimethoxybenzoic acid	Phenolic Acids	181.1 $[M-H]^-$	136.7	-49	-10.9	-10.6	-9	4.5
Acetylphloroglucinol	Phenolic Acids	167.0 $[M-H]^-$	122.8	-49	-10.9	-23	-11	5.05
Salicylic acid	Phenolic Acids	137.0 $[M-H]^-$	92.6	-49	-10.9	-25	-7	5.54
trans-2-Hydroxycinnamic acid	Phenolic Acids	163.0 $[M-H]^-$	119.2	-49	-10.9	-15	-26	6.14
Caffeic acid dimethyl ether	Phenolic Acids	207.0 $[M-H]^-$	102.7	-49	-10.9	-19	-16	7.34
3-Methoxyhydrocinnamic acid	Phenolic Acids	179.1 $[M-H]^-$	119.9	-49	-10.9	-17.5	-13	7.48
Gallic acid	Phenolic Acids	169.0 $[M-H]^-$	124.9	-49	-10.9	-21	-9	0.52
3,5-Dihydroxybenzoic acid	Phenolic Acids	153.0 $[M-H]^-$	108.8	-49	-10.9	-15	-9	1.02

Vanillic acid	Phenolic Acids	167.0 [M-H]-	151.8	-49	-10.9	-18	-14	3.07
Nordihydroguaiaretic Acid	Phenolic Acids	301.1 [M-H]-	121.6	-49	-10.9	-34.5	-23	8.25
Terephthalic acid	Phenolic Acids	165.0 [M-H]-	120.5	-49	-10.9	-14	-12	3.19
4-Acetylresorcinol	Phenolic Acids	151.0 [M-H]-	91.0	-49	-10.9	-25.5	-20	5.09
Rosmarinic acid	Phenolic Acids	359.1 [M-H]-	160.6	-49	-10.9	-16.5	-11	7.48
Caffeic acid phenethyl ester	Phenolic Acids	283.09 [M-H]-	134.8	-49	-10.9	-29	-29	8.35
2,3,4-Trihydroxybenzoic acid	Phenolic Acids	169.0 [M-H]-	150.8	-49	-10.9	-16.5	-16	1.15
2,4-Dihydroxybenzoic Acid	Phenolic Acids	153.0 [M-H]-	109.1	-49	-10.9	-17	-8	2.66
3-Hydroxybenzoic acid	Phenolic Acids	137.0 [M-H]-	92.8	-49	-10.9	-17	-27	2.74
Coniferyl Alcohol	Phenolic Acids	179.1 [M-H]-	145.6	-49	-10.9	-18	-5	4.5
m-Coumaric acid	Phenolic Acids	163.04 [M-H]-	118.8	-49	-10.9	-18	-19	5.38
2-Acetylresorcinol	Phenolic Acids	151.04 [M-H]-	134.6	-49	-10.9	-22	-7	5.96
3,4,5-Trimethoxycinnamic acid	Phenolic Acids	237.1 [M-H]-	103.2	-49	-10.9	-19	-27	7.61
Kaempferol	Flavonoids	285.0 [M-H]-	197.8	-56	-8.5	-36	-9	8.09
Phloretin	Flavonoids	273.1 [M-H]-	166.9	-56	-8.5	-24	-15	8
7,8-Dihydroxyflavone	Flavonoids	317.0 [M-H]-	178.8	-56	-8.5	-28	-19	7.48
Myricetin	Flavonoids	389.1 [M-H]-	148.8	-56	-8.5	-38	-31	8.5
Polydatin	Flavonoids	301.0 [M-H]-	150.3	-56	-8.5	-24	-5	7.88
Quercetin	Flavonoids	285.08 [M-H]-	184.5	-56	-8.5	-40	-13	8.09
Acacetin	Flavonoids	269.0 [M-H]-	194.6	-56	-8.5	-35	-34	8.21
Baicalein	Flavonoids	223.1 [M-H]-	91.6	-56	-8.5	-38	-8	8.33
4'-Hydroxychalcone	Flavonoids	289.1 [M-H]-	252.9	-56	-8.5	-19	-17	7.9
(+)-Catechin (Hydrate)	Flavonoids	301.0 [M-H]-	150.3	-56	-8.5	-24	-5	7.88
Mangiferin	Flavonoids	421.1 [M-H]-	300.9	-56	-8.5	-27	-14	5.21
(+)-Taxifolin	Flavonoids	303.0 [M-H]-	285.0	-56	-8.5	-17	-20	5.97
Diosmetin	Flavonoids	299.0 [M-H]-	284.0	-56	-8.5	-28	-21	8.16
Morin	Flavonoids	301.0 [M-H]-	150.5	-56	-8.5	-31	-32	7.85
(-)-Epigallocatechin gallate hydrate	Flavonoids	457.1 [M-H]-	168.9	-56	-8.5	-16	-8	4.96
Chrysin	Flavonoids	253.1 [M-H]-	142.8	-56	-8.5	-41	-41	8.42
(+/-)-Naringenin	Flavonoids	271.1 [M-H]-	118.8	-56	-8.5	-28	-14	7.98
Baicalin	Flavonoids	445.1 [M-H]-	268.5	-56	-8.5	-27	-18	8.05
Resveratrol	Flavonoids	227.0 [M-H]-	184.4	-56	-8.5	-22	-9	7.34
Luteolin	Flavonoids	285.0 [M-H]-	133.0	-56	-8.5	-41	-37	7.3
Hesperidin	Flavonoids	609.2 [M-H]-	300.7	-56	-8.5	-31	-31	7.63
Fisetin	Flavonoids	285.0 [M-H]-	134.5	-56	-8.5	-27	-14	7.55
(-)-Epicatechin	Flavonoids	289.1 [M-H]-	252.8	-56	-8.5	-22	-22	7.75
Oxyresveratrol	Flavonoids	243.0 [M-H]-	200.3	-56	-8.5	-25	-6	6.64

Apigenin	Flavonoids	269.0 $[M-H]^-$	116.4	-56	-8.5	-47	-7	8.1
trans-Pterostilbene	Flavonoids	255.1 $[M-H]^-$	239.5	-56	-8.5	-27	-6	8.46
Rutin Hydrate	Flavonoids	609.1 $[M-H]^-$	299.7	-56	-8.5	-47	-9	7.27
Phloridzin	Flavonoids	435.1 $[M-H]^-$	272.5	-56	-8.5	-20	-37	7.51
Daidzein	Flavonoids	253.1 $[M-H]^-$	207.5	-56	-8.5	-35	-28	7.74
Hesperetin	Flavonoids	301.1 $[M-H]^-$	163.4	-56	-8.5	-39	-11	8.05
Puerarin	Flavonoids	415.1 $[M-H]^-$	294.9	-56	-8.5	-30	-12	5.09
Isoliquiritigenin	Flavonoids	255.1 $[M-H]^-$	119.0	-56	-8.5	-27	-33	8.13
Piceatannol	Flavonoids	243.06 $[M-H]^-$	158.3	-56	-8.5	-26	-13	6.35
Biochanin A	Flavonoids	283.1 $[M-H]^-$	267.7	-56	-8.5	-26	-10	8.42
Formononetin	Flavonoids	267.1 $[M-H]^-$	251.5	-56	-8.5	-28	-4	8.21
Diosmin	Flavonoids	607.2 $[M-H]^-$	298.4	-56	-8.5	-22	-21	7.67
Naringin dihydrochalcone	Flavonoids	581.18 $[M-H]^-$	273.0	-56	-8.5	-38	-10	7.75
Equol	Flavonoids	243.0 $[M-H]^-$	200.8	-56	-8.5	-27	-19	6.33
Genistein	Flavonoids	269.0 $[M-H]^-$	132.7	-56	-8.5	-34	-16	7.98

[a]DP= declustering potential;EP= entrance potential [c]CE= collision energy; [d]CXP= Collision cell exit potential; [e]RT= retention time.

Table S2. Linearity and sensitivity data.

**Metabolite**	**Metabolite Class**	**Linearity range (g/mL)**	**r^2**	**LOD**	**LOQ**	**Curve equation**
punicalagin anomer	Phenolic compound	1.00-40.0	0.9986	1	2.5	y = 6143.6x – 7350.7
punicalagin anomer b	Phenolic compound					
o-Anisic Acid	Phenolic Acids	0.03–0.63	0.998	0.01	0.03	y = 3E+06x + 44109
2,3-Dihydroxybenzoic acid	Phenolic Acids	0.03–5.00	0.997	0.006	0.03	y = 2E+06x + 189509
Chlorogenic acid	Phenolic Acids	0.03–5.00	0.997	0.002	0.005	y = 1E+06x + 87762
Syringic acid	Phenolic Acids	0.63–10.0	0.998	0.31	0.63	y = 17964x - 1183.6
p-Coumaric acid	Phenolic Acids	0.03–2.50	0.998	0.005	0.02	y = 2E+06x + 122038
m-Hydrocoumaric acid	Phenolic Acids	0.31–10.0	0.999	0.03	0.31	y = 943964x + 150482
Ferulic acid	Phenolic Acids	0.03–10.0	0.999	0.002	0.03	y = 559017x + 57790
Sinapic acid	Phenolic Acids	0.03–10.0	0.999	0.002	0.005	y = 222015x + 3754.9
Aspirin	Phenolic Acids	0.03–10.0	0.998	0.002	0.007	y = 405391x - 67347
trans-Cinnamic acid	Phenolic Acids	2.50–10.0	0.995	1.50	2.50	y = 36651x - 21342
4-Hydroxybenzoic acid	Phenolic Acids	0.03–1.25	0.998	0.006	0.03	y = 2E+06x + 26546
2,6-Dihydroxybenzoic acid	Phenolic Acids	0.003–2.50	0.997	0.001	0.003	y = 4E+06x + 164093
Dihydrocaffeic acid	Phenolic Acids	0.03–5.00	0.999	0.002	0.007	y = 538677x + 27979
Caffeic acid	Phenolic Acids	0.31–2.50	0.997	0.03	0.12	y = 1E+06x + 153979
Phloretic acid	Phenolic Acids	Not quantifiable in the selected linearity range				
Hydroferulic acid	Phenolic Acids	0.03–5.00	0.999	0.001	0.003	y = 270948x + 1777.7
Ellagic acid dihydrate	Phenolic Acids	0.03–1.25	0.997	0.007	0.03	y = 99817x + 3393
5-Methoxysalicylic acid	Phenolic Acids	0.03–5.00	0.999	0.001	0.03	y = 3E+06x + 126683
Catechol	Phenolic Acids	0.03–2.50	0.996	0.001	0.004	y = 1E+06x + 53882
Gentisic acid	Phenolic Acids	0.003–1.25	0.999	0.001	0.003	y = 1E+06x + 6905.2
4-Acetocatechol	Phenolic Acids	0.03–2.50	0.996	0.007	0.03	y = 284650x + 23702
4-Methylcatechol	Phenolic Acids	0.31–10.0	0.999	0.03	0.14	y = 7528x + 825.75
2,6-Dimethoxybenzoic acid	Phenolic Acids	1.25 – 10.0	0.996	0.63	1.25	y = 49710x + 38212
Acetylphloroglucinol	Phenolic Acids	0.03–2.50	0.994	0.004	0.03	y = 2E+06x + 100214
Salicylic acid	Phenolic Acids	0.03–10.0	0.999	0.002	0.03	y = 3E+06x + 402246
trans-2-Hydroxycinnamic acid	Phenolic Acids	0.03–5.00	0.998	0.003	0.03	y = 2E+06x + 193871

Caffeic acid dimethyl ether	Phenolic Acids	0.31–10.0	0.998	0.03	0.08	y = 42692x - 4380
3-Methoxyhydrocinnamic acid	Phenolic Acids	0.31–10.0	0.997	0.03	0.11	y = 16741x - 2051.4
Gallic acid	Phenolic Acids	0.03–2.50	0.999	0.005	0.03	y = 1E+06x + 37728
3,5-Dihydroxybenzoic acid	Phenolic Acids	0.31–5.00	0.998	0.03	0.31	y = 695154x + 108182
Vanillic acid	Phenolic Acids	0.31–10.0	0.999	0.03	0.01	y = 25878x - 747.51
Nordihydroguaiaretic Acid	Phenolic Acids	0.03–2.50	0.999	0.001	0.002	y = 2E+06x + 21094
Terephthalic acid	Phenolic Acids	0.03–2.50	0.999	0.01	0.03	y = 1E+06x + 35990
4-Acetylresorcinol	Phenolic Acids	0.03–2.50	0.999	0.001	0.003	y = 906372x + 17474
Rosmarinic acid	Phenolic Acids	0.03–5.00	0.999	0.0005	0.001	y = 1E+06x - 3641.7
Caffeic acid phenethyl ester	Phenolic Acids	0.03–2.50	0.997	0.001	0.03	y = 5E+06x + 215577
2,3,4-Trihydroxybenzoic acid	Phenolic Acids	0.03–0.60	0.999	0.001	0.003	y = 874629x + 5590.4
2,4-Dihydroxybenzoic Acid	Phenolic Acids	0.03–5.00	0.998	0.01	0.03	y = 928863x + 55270
3-Hydroxybenzoic acid	Phenolic Acids	0.31–5.00	0.998	0.03	0.31	y = 640365x + 43840
Coniferyl Alcohol	Phenolic Acids	Not quantifiable in the selected linearity range				
m-Coumaric acid	Phenolic Acids	0.03–2.50	0.999	0.001	0.03	y = 2E+06x + 80336
2-Acetylresorcinol	Phenolic Acids	0.03–1.25	0.996	0.002	0.005	y = 3E+06x + 99822
3,4,5-Trimethoxycinnamic acid	Phenolic Acids	0.03–10.0	0.998	0.0003	0.03	y = 105739x + 13535
Kaempferol	Flavonoids	0.31–2.50	0.996	0.03	0.1	y = 31889x + 313.64
Phloretin	Flavonoids	0.03–2.50	0.998	0.0004	0.001	y = 6E+06x + 181590
Myricetin	Flavonoids	0.31–5.00	0.995	0.05	0.15	y = 669096x - 127523
Polydatin	Flavonoids	Not quantifiable in the selected linearity range				
Quercetin	Flavonoids	0.03–2.50	0.995	0.003	0.03	y = 232809x + 10141
Acacetin	Flavonoids	0.03–2.50	0.996	0.004	0.03	y = 141397x + 7498.9
Baicalein	Flavonoids	0.03–5.00	0.998	0.004	0.03	y = 244740x - 4360.9
4'-Hydroxychalcone	Flavonoids	0.03–2.50	0.995	0.002	0.03	y = 687940x + 15160
(+)-Catechin (Hydrate)	Flavonoids	0.31–1.25	0.997	0.03	0.06	y = 98018x + 18549
Mangiferin	Flavonoids	0.03–5.00	0.999	0.001	0.003	y = 1E+06x + 84377
(+)-Taxifolin	Flavonoids	0.03–5.00	0.999	0.002	0.005	y = 2E+06x + 50504
Diosmetin	Flavonoids	0.03–1.25	0.997	0.002	0.005	y = 6E+06x + 112067
Morin	Flavonoids	0.03–5.00	0.997	0.002	0.005	y = 717491x + 68919
(-)-Epigallocatechin gallate hydrate	Flavonoids	0.03–10.0	0.998	0.008	0.03	y = 566243x - 71657
Chrysin	Flavonoids	0.03–5.00	0.996	0.0006	0.03	y = 598355x + 56081
(+/-)-Naringenin	Flavonoids	0.03–5.00	0.996	0.0005	0.001	y = 2E+06x - 15413
Baicalin	Flavonoids	0.03–2.50	0.997	0.001	0.003	y = 1E+06x - 10656
Resveratrol	Flavonoids	0.03–10.0	0.998	0.009	0.03	y = 46605x + 1953.5
Luteolin	Flavonoids	Not quantifiable in the selected linearity range				
Hesperidin	Flavonoids	0.03–10.0	0.996	0.002	0.006	y = 433221x + 1285.5

Fisetin	Flavonoids	0.03–5.00	0.996	0.002	0.03	y = 976215x + 46400
(-)-Epicatechin	Flavonoids	0.03–1.25	0.996	0.01	0.03	y = 141591x + 674.48
Oxyresveratrol	Flavonoids	0.03–10.0	0.998	0.008	0.03	y = 30531x + 1319
Apigenin	Flavonoids	0.03–1.25	0.994	0.001	0.005	y = 763274x - 26083
trans-Pterostilbene	Flavonoids	0.31–10.0	0.996	0.007	0.03	y = 52998x - 1462.5
Rutin Hydrate	Flavonoids	0.03–5.00	0.995	0.002	0.005	y = 1E+06x + 15351
Phloridzin	Flavonoids	0.03–5.00	0.997	0.0005	0.002	y = 437141x - 8944
Daidzein	Flavonoids	0.03–2.50	0.995	0.005	0.03	y = 247718x + 22978
Hesperetin	Flavonoids	0.03–5.00	0.996	0.0005	0.001	y = 619584x - 14267
Puerarin	Flavonoids	0.03–5.00	0.998	0.0005	0.002	y = 2E+06x - 1073
Isoliquiritigenin	Flavonoids	Not quantifiable in the selected linearity range				
Piceatannol	Flavonoids	0.03–10.0	0.997	0.009	0.03	y = 105690x + 17925
Biochanin A	Flavonoids	0.03–1.25	0.993	0.0004	0.001	y = 1E+07x + 287361
Formononetin	Flavonoids	0.03–0.63	0.999	0.00001	0.003	y = 2E+07x + 33838
Diosmin	Flavonoids	0.03–5.00	0.997	0.002	0.008	y = 221995x - 1498.1
Naringin dihydrochalcone	Flavonoids	0.03–10.0	0.999	0.004	0.03	y = 418240x + 42251
Equol	Flavonoids	0.03–2.50	0.997	0.002	0.005	y = 727655x + 19681
Genistein	Flavonoids	0.03–5.00	0.994	0.01	0.03	y = 486314x - 46341

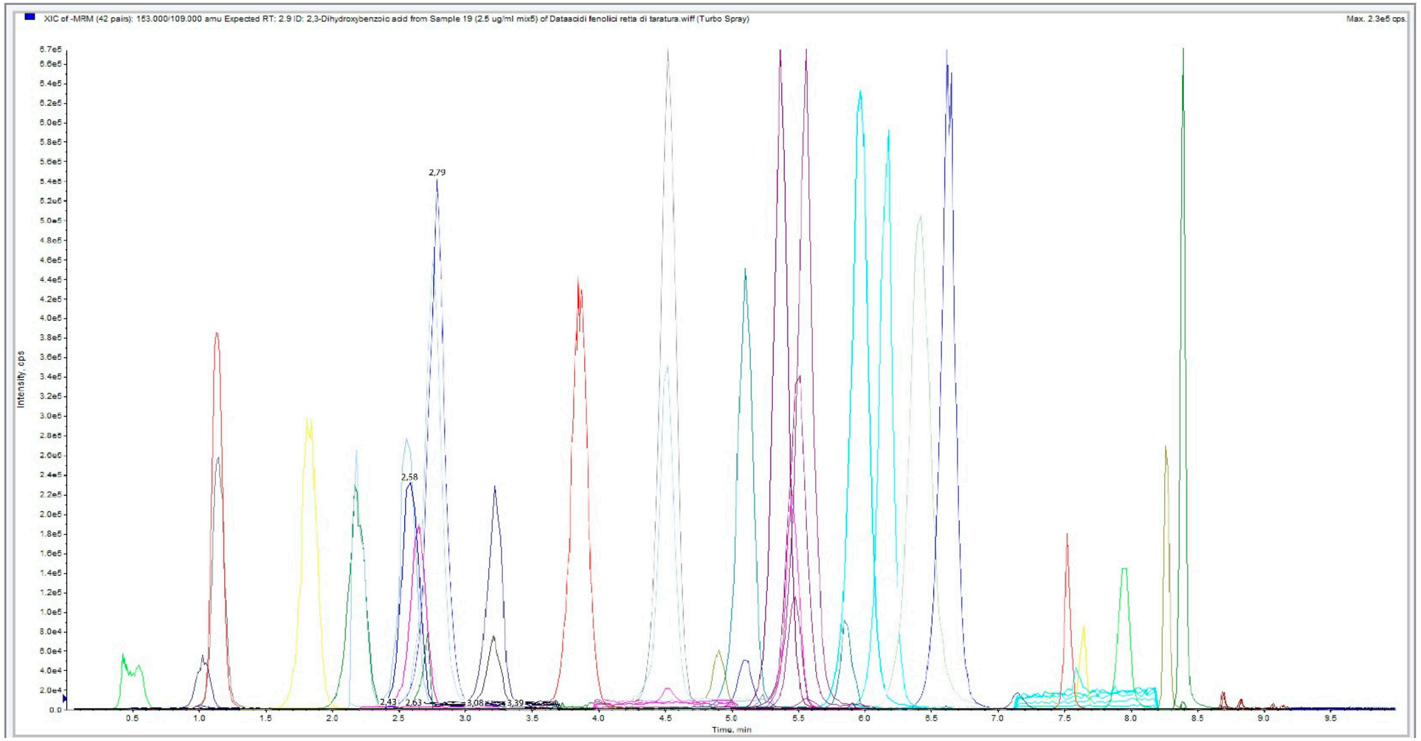

Figure S1. Extract Ion Chromatogram (XIC) of Multi Reaction Monitoring (MRM) of the optimized 43 phenolic acids and punicalagin.

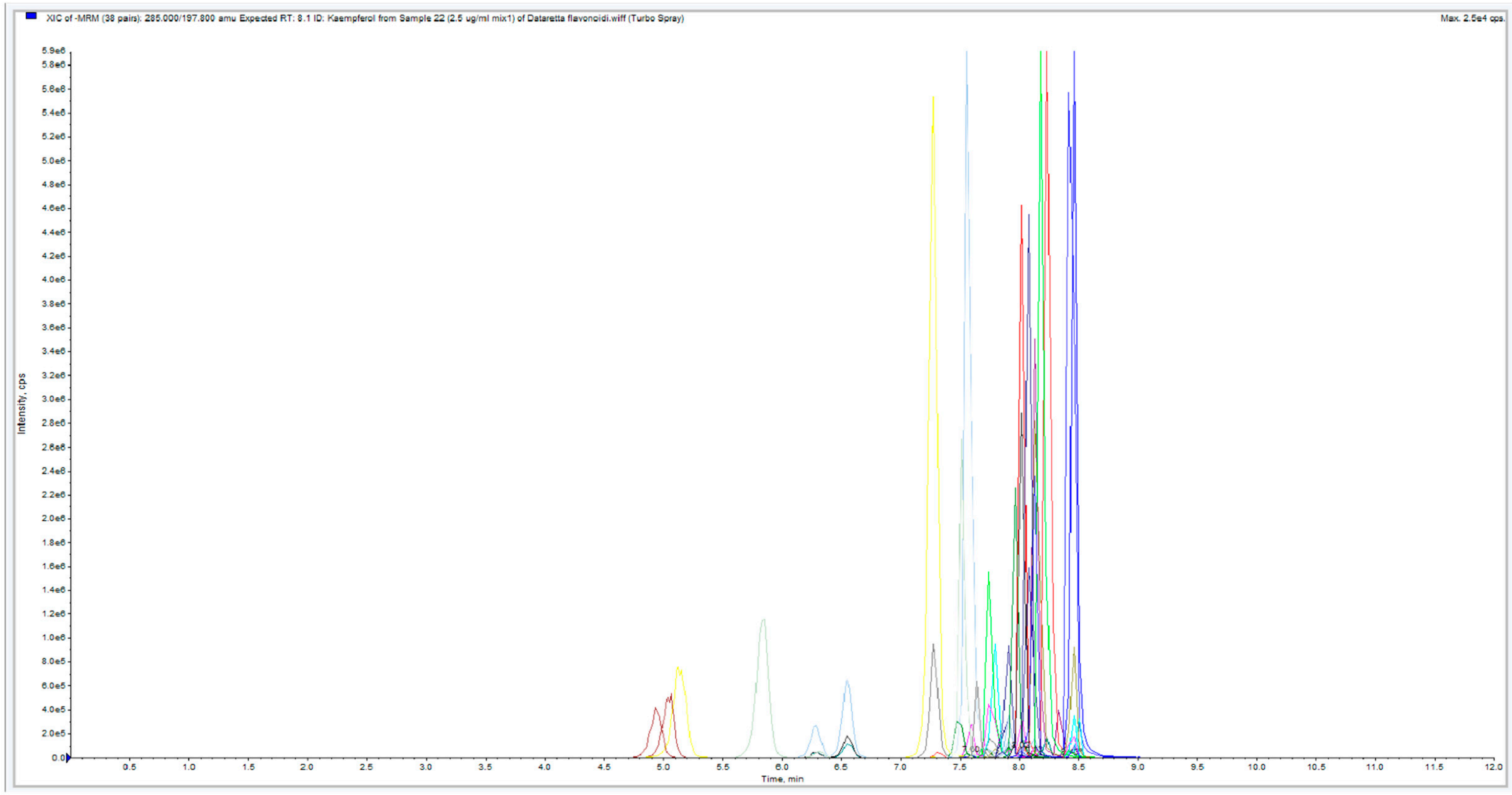

Figure S2. Extract Ion Chromatogram (XIC) of Multi Reaction Monitoring (MRM) of the optimized 38 flavonoids.

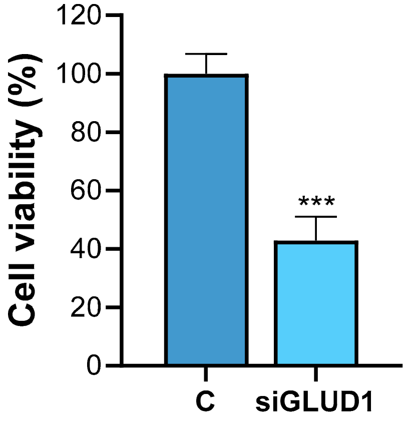

Figure S3. *GLUD1* knockdown reduces CCLP1 cell viability. CCLP1 cells were transfected with a siRNA targeting human *GLUD1* (siGLUD1) or a control scramble siRNA (C). Seventy-two hours later, cell viability was assessed. Data represent means ± S.D. of three independent experiments; differences were significant according to Student’s t-test (***$p < 0.001$).
